# A perspective on arthroscopic treatment for anterior ankle impingement syndrome: clinical research insights

**DOI:** 10.3389/fsurg.2025.1613472

**Published:** 2026-01-21

**Authors:** Long-Ze Zong, Yong Feng, Dong-Yu Bai

**Affiliations:** Department of Orthopedics, Affiliated Hospital of Yan‘an University, Yan'an, China

**Keywords:** anterior ankle impingement syndrome, arthroscopic treatment, ankle disorder, insight, perspective

## Abstract

**Introduction:**

Anterior ankle impingement syndrome (AAIS) is a degenerative condition that causes anterior ankle pain and limited dorsiflexion, especially in athletes. It results from either osseous (osteophytes) or soft tissue (synovial hypertrophy, fibrosis) pathology.

**Methods:**

Although conservative treatments offer temporary relief, arthroscopic surgery has become the preferred approach due to its minimally invasive technique and surgical precision.

**Results:**

Current evidence shows 80%–90% success rates, with significant improvements in visual analog scale scores (mean reduction of 4.1 points) and American orthopedic foot & ankle society scores (mean increase of 28 points), along with low complication rates (2%–7%). However, outcomes are closely linked to the severity of pre-existing osteoarthritis, with 93% success in non-arthritic joints compared to 53% in cases with moderate osteoarthritis. Key research limitations include heterogeneous study designs, small sample sizes, and a lack of long-term data (only 18.6% of studies report ≥5-year follow-up).

**Discussion:**

Future research should focus on standardizing outcome measures, assessing the cost-effectiveness of advanced techniques, and establishing evidence-based protocols for patient selection and rehabilitation. These efforts will help optimize surgical decision-making and enhance long-term outcomes for patients with AAIS.

## 1 Introduction

Anterior ankle impingement syndrome (AAIS) causes chronic ankle disorder characterized by anterior ankle pain and restricted dorsiflexion, primarily affecting athletes, dancers, and others performing repetitive high-impact ankle movements (1–3). The condition arises from either bony impingement (e.g., tibial or talar osteophytes) or soft tissue impingement (e.g., synovial hypertrophy, thickened ligament, or fibrosis) (1–3). Epidemiological data indicate that AAIS affects approximately 20%–40% of soccer players and a higher proportion of ballet dancers, severely limiting mobility and quality of life (4).

Treatment options range from conservative therapies (e.g., physical therapy, non-steroidal anti-inflammatory drugs, corticosteroid injections) to open surgery (5, 6). Although helpful for some, conservative management often fails with structural abnormalities (e.g., prominent osteophytes or ligamentous hypertrophy), leading to frequent symptom recurrence (5, 6). While open surgery effectively treats pathological structures, it risks extensive tissue injury, slow recovery, scarring, and joint stiffness (7). Recently, arthroscopic techniques have become preferred in foot/ankle surgery (8–10). Arthroscopy provides smaller incisions, better visualization, faster recovery, and precise debridement with less tissue injury than open surgery (8–10).

Despite its benefits, arthroscopy's optimal use and long-term results remain debated (8–11). Some report better outcomes for bony vs. soft tissue impingement, whereas others like recurrence to incomplete debridement or poor rehabilitation (10, 11). Moreover, high-quality long-term data are lacking, preventing firm conclusions about durability (12).

Our perspective offers a focused examination of arthroscopic treatment for AAIS, integrating clinical experience with published findings to highlight key technical considerations, treatment outcomes, and associated complications. Drawing from both established data and current practice patterns, we identify critical areas where evidence remains limited while offering practical guidance for clinicians. This study aims to bridge the gap between existing research and real-world clinical application, presenting a balanced view of arthroscopic management for AAIS that may help refine treatment approaches and decision-making.

## 2 Study retrieval and selection

**Figure 1:**
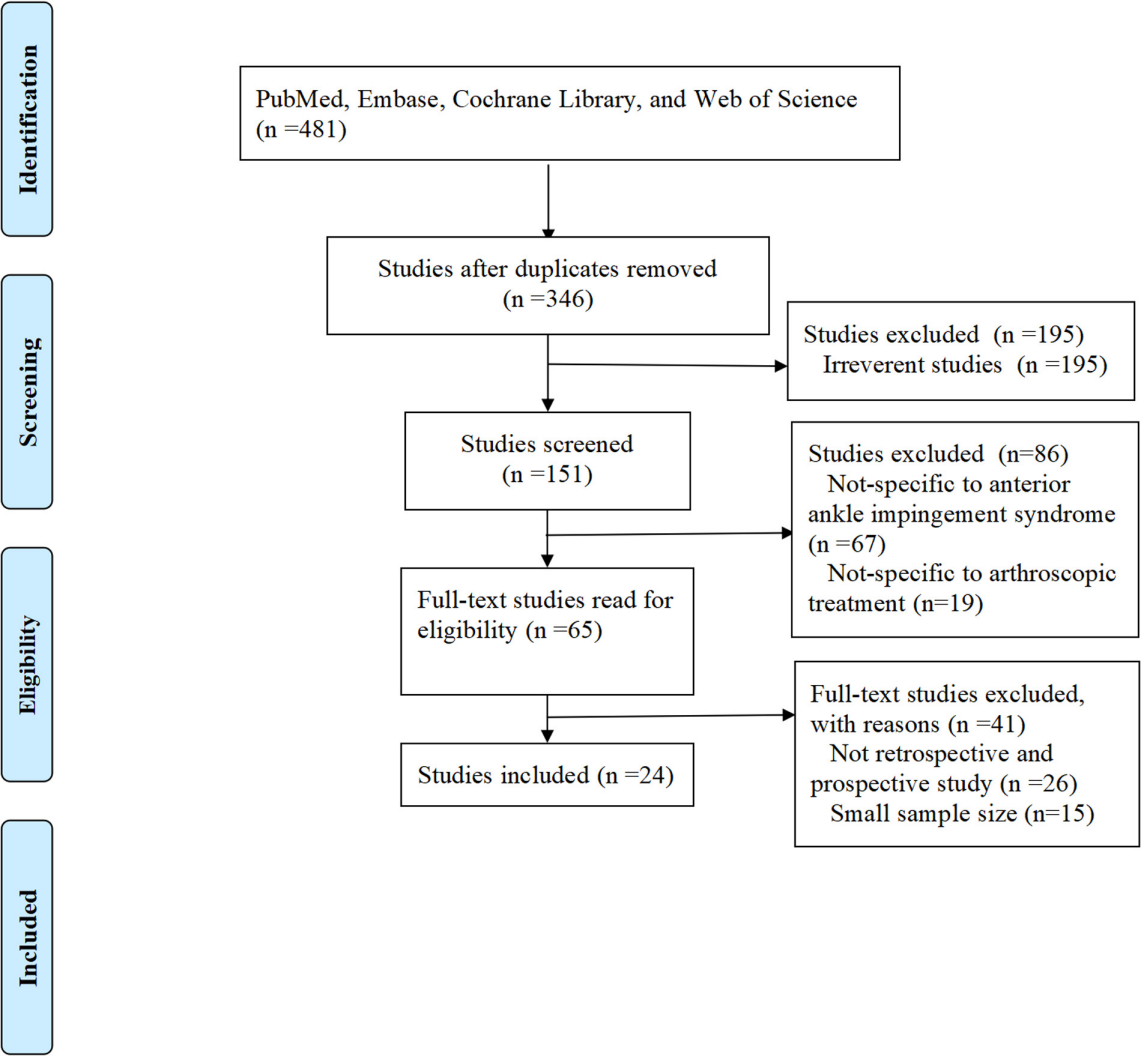
Flowchart of study selection.

We systematically searched four major medical databases (including PubMed, Embase, Cochrane Library, and Web of Science) for studies on arthroscopic AAIS treatment. The search used key terms such as “anterior ankle impingement,” “ankle arthroscopy,” and “surgical treatment,” without publication date or language restrictions. After duplicate removal, 346 unique records remained. Two authors independently screened titles/abstracts using predefined eligibility criteria: (1) clinical studies (prospective or retrospective designs); (2) patients with confirmed AAIS; (3) primary arthroscopic treatment; and (4) reported outcome measures such as pain scales, functional scores, complications, or recurrence rates. We excluded non-clinical studies, reviews, small case reports (<10 patients), and duplicates. After screening, 322 records were excluded. All 24 remaining articles met inclusion criteria after full-text review and were analyzed (Figure 1).

## 3 Pathophysiology and diagnosis of AAIS

### 3.1 Etiology and classification

AAIS is an important clinical condition with distinct causes and diagnostic challenges (2, 5–7). Its pathophysiology includes two main types: osseous impingement (from tibial/alar osteophytes caused by repetitive microtrauma in athletes) and soft tissue impingement (featuring synovial proliferation, ligament thickening, or fibrosis) that typically follows ankle trauma (13, 14).

Current classification systems offer important diagnostic guidance. The Scranton-McDermott system stages AAIS by osteophyte size and location, while the van Dijk classification focuses on soft tissue patterns that guide surgical decisions (15, 16).

### 3.2 Clinical presentation and imaging evaluation

AAIS typically presents with anterior ankle pain worsened by dorsiflexion, tenderness, limited motion, and sometimes mechanical symptoms such as catching or locking (1, 2, 5–9).

Diagnosis requires a multimodal approach beginning with weight-bearing radiographs for initial osteophyte assessment. When indicated, advanced imaging modalities provide additional value: magnetic resonance imaging (MRI) offers superior soft tissue resolution and detects early bone marrow changes; dynamic ultrasonography enables real-time functional assessment; and computed tomography (CT) reconstruction provides detailed osseous architecture evaluation (6, 7, 13, 14).

Notably, approximately 15%–20% of cases show clinically significant discordance between imaging findings and symptom severity, necessitating comprehensive clinical correlation and sometimes diagnostic injections for definitive pain source identification (13, 14, 17). This diagnostic approach emphasizes integrating clinical examination with targeted imaging to optimize accuracy and guide therapeutic interventions.

## 4 Technical aspects of arthroscopic surgery for AAIS

### 4.1 Indications and contraindications

Arthroscopic intervention for AAIS is principally indicated for patients who demonstrate persistent symptoms after at least three months of conservative management (including physical therapy, pharmacologic intervention, and intra-articular injections), accompanied by functional impairment, radiographically confirmed osteophytes (Scranton-McDermott type II-IV), or soft tissue impingement (van Dijk classification), or when both diagnosis and treatment are required (10, 15, 16, 18). It is also relatively indicated for high-performance athletes seeking a faster return to sport.

Contraindications require careful assessment, including advanced ankle osteoarthritis (Takakura stage III or higher) with marked joint space narrowing, active local or systemic infection, severe peripheral vascular disease, and poor patient compliance (19, 20). Notably, diabetes and smoking are relative contraindications, but patients should be informed of the increased risk of poor wound healing and infection (20).

### 4.2 Surgical technique

Arthroscopic treatment of anterior ankle impingement syndrome typically follows a standardized dual-portal approach through anteromedial and anterolateral access points (16). The patient is positioned supine with a proximal thigh tourniquet, and the ankle extended beyond the operating table to optimize access to the joint. Accurate portal placement is essential—the anterolateral portal is established 1 cm proximal to the joint line, lateral to the peroneus tertius tendon, while the anteromedial portal is created 1 cm proximal and medial to the tibialis anterior tendon, ensuring protection of the superficial peroneal and saphenous nerves (8–12).

The procedures begins with a systematic exploration of the joint, following the sequence: medial gutter, central compartment, and lateral gutter (7–10, 12, 15, 19). Surgeons use 3.5–4.0 mm arthroscopes with compatible shavers, maintaining joint distension with controlled irrigation (30–40 mmHg) and intermittent tourniquet inflation in 60-min cycles (7–10, 12). Osteophytes are removed using curved osteotomes or burrs, following a peripheral-to-central approach to preserve intact articular cartilage (7–10). Synovial debridement is performed using radiofrequency ablation or mechanical resection, focusing on hypertrophic areas while preserving healthy synovium. Ligamentous structures are managed conservatively, trimming only fibrotic portions to maintain joint stability (7–10, 12, 15). Careful hemostasis and periodic tourniquet release throughout the procedure minimize soft tissue trauma and enhance visualization.

### 4.3 Intraoperative challenges and solutions

Several technical challenges may occur during arthroscopic AAIS management, each necessitating tailored strategies. Suboptimal visualization, especially in cases of extensive synovitis or fibrosis, can be managed with a stepwise approach: increasing irrigation pressure (up to 50 mmHg), switching to a 2.7 mm arthroscope if necessary, using radiofrequency ablation to achieve hemostasis, and, if needed, creating additional portals to ensure procedural safety (21, 22).

Neurovascular protection is critical, as the anterolateral portal poses a 3.6% risk of superficial peroneal nerve injury, and the anteromedial portal requires safeguarding the saphenous neurovascular bundle (23–25). A standardized safety protocol involves preoperative mapping and skin marking of superficial neurovascular structures, use of blunt dissection for portal creation, and continuous visualization of instrument tips during all intra-articular steps (23–25).

Preventing postoperative adhesion involves several evidence-based strategies: complete hemostasis before wound closure, early passive range-of-motion exercises (within 24 h), use of hyaluronate-based anti-adhesion barriers in high-risk cases, and application of continuous passive motion devices for 2–3 weeks in patients with a history of arthrofibrosis (26, 27). These combined measures address common intraoperative challenges and help ensure both procedural success and favorable long-term outcomes (26, 27).

## 5 Clinical outcomes and evidence

### 5.1 Literature review

Recent prospective and retrospective studies have consistently confirmed the effectiveness of arthroscopic surgery in treating AAIS (10, 11, 16, 28–48) (Table 1). Notably, Nihal et al. (28) reported that 75% (9/12) of dancers resumed full activity within an average of seven weeks postoperatively, demonstrating the procedure's value in high-demand populations. Cuellar-Avaroma et al. (29) reported significant improvements in a 52-patient cohort, with visual analog scale (VAS) pain scores decreasing from 5.75 to 0.98 and American orthopedic foot & ankle society (AOFAS) scores increasing from 73.65 to 92.98. Additionally, 44.23% of patients returned to pre-injury sports within 4–7 months. Wang et al. (30) compared dual- and triple-portal arthroscopy, finding no significant differences in AOFAS scores (76.18 vs. 79.18) or dorsiflexion (21.36° vs. 20.45°), although the triple-portal approach was more beneficial in severe cases.

**Table 1:** Summarizing the key details of studies on arthroscopic surgery for AAIS.

Study	Patients	Publication type	Treatment	Main findings
Tol et al. (10)	57 patients with AAIS	Prospective study	Arthroscopic surgery	- Excellent/good results in all patients without OA. - 77% of grade-I OA patients had excellent/good results despite osteophyte recurrence. - 53% of grade-II OA patients had excellent/good results.
Baums et al. (11)	26 patients with AAIS	Prospective study	Arthroscopic surgery	- Karlsson score improved from 66 to 92 (*P* < 0.05). - Tegner score improved from 3 to 8. - 96% patient satisfaction. - No difference between soft-tissue vs. bony impingement outcomes.
van Dijk et al. (16)	62 patients with AAIS	Prospective study	Arthroscopic surgery	- 90% good/excellent results without OA vs. 50% with OA. - Medial spurs had better outcomes. - Shorter pain duration (<2 yrs) predicted better results.
Nihal et al. (28)	11 patients with AAIS	Retrospective study	Arthroscopic surgery	- 82% returned to dance (7 weeks). - 2 reoperations (recurrent spur/scar tissue). - Minimal morbidity.
Cuellar-Avaroma et al. (29)	52 patients with AAIS	Prospective study	Arthroscopic surgery	- Pain scale improved from 5.75 → 0.98. - AOFAS improved from 73.65 → 92.98. - 44% returned to sports (4–7 months).
Wang et al. (30)	52 patients with AAIS	Retrospective study	Arthroscopic surgery	- No significant difference in AOFAS/VAS/dorsiflexion between groups. - 3-portal suggested for advanced impingement.
Murawski et al. (31)	41 patients with AAIS	Retrospective study	Arthroscopic surgery	- 93% patient satisfaction. - AOFAS scores improved from 62.83 to 91.17 (*P* < 0.001). - 97% returned to prior sports level. - 7% complication rate.
Jerosch et al. (32)	35 patients with AAIS	Retrospective study	Arthroscopic surgery	- Pain improved significantly (*P* < 0.05). - Only 26% returned to prior sports level. - 17% had temporary neurological complications. - Hypermobile ankles had worse outcomes.
Branca et al. (33)	58 patients with AAIS	Retrospective study	Arthroscopic surgery	- Postoperative McGuire scores: 37 good, 13 fair, 8 poor. - Recurrence in severe cases (McDermott stage III/IV). No major complications.
Rouvillain et al. (34)	24 patients with AAIS	Retrospective study	Arthroscopic surgery	- 92% excellent results (Kitaoka score). - No major complications (2 transient hypoesthesias, resolved by 6 months). - No distraction needed (simple dorsiflexion sufficient).
Akseki et al. (35)	21 patients with AAIS	Prospective study	Arthroscopic surgery	- 81% (17/21) good/excellent results. - 90% patient satisfaction. - 2 poor results due to neuromas (resolved with steroids).
Arnold (36)	32 patients with AAIS	Retrospective study	Arthroscopic surgery	- 81% (26/32) good/excellent results (West Point Ankle Score ≥80). - Worse outcomes with severe chondral lesions. - Effective for synovium/fibrous bands (type I) and tibial spurs (types II–III).
Yang et al. (37)	22 patients with AAIS	Prospective study	Arthroscopic surgery	- AAI group had worse pre-op scores but no difference post-op. - Dorsiflexion improved (13° → 26°). - No osteophyte recurrence.
Devgan et al. (38)	14 patients with AAIS	Prospective study	Arthroscopic surgery	- Both groups improved (AOFAS: 50.5 → 85.71). - Soft tissue group had slightly better range of motion and faster return to sports.
Ferkel et al. (39)	31 patients with AAIS	Retrospective study	Arthroscopic surgery	- 84% (26/31) excellent/good results. - MRI useful for diagnosing synovial thickening. - Average return to sports: 6 weeks.
Hassan (40)	23 patients with AAIS	Prospective study	Arthroscopic surgery	- AOFAS improved from 34 to 89. - 91% (21/23) excellent/good results. - 96% would repeat surgery.
Koczy et al. (41)	22 patients with AAIS	Prospective study	Arthroscopic surgery	- AOFAS improved from 75.4 to 92 at 12 months. - 1 case of temporary nerve palsy. - Effective for post-sprain/fracture impingement.
Walsh et al. (42)	46 patients with AAIS	Prospective study	Arthroscopic surgery	- Foot Functional Index improved (20.5 → 2.7). - Minimal dorsiflexion improvement (24.7° → 27°). - 84% had osteophyte recurrence but no functional decline.
Liu et al. (43)	55 patients with AAIS	Retrospective study	Arthroscopic surgery	- 87% good/excellent results. - 98% patient satisfaction. - 84% returned to sports.
Brennan et al. (44)	41 patients with AAIS	Retrospective study	Arthroscopic surgery	- Visual-Analogue-Scale Foot and Ankle scores improved from 44.5 to 78.3 (*P* < 0.0001). - 83% good/excellent results (Meislin criteria). - MRI underreported synovitis/impingement.
Cavallo et al. (45)	600 patients with AAIS	Retrospective study	Arthroscopic surgery	- 80% good/excellent results for impingement. - AOFAS scores improved postoperatively. - Effective for ligament injuries, fractures, and osteochondral lesions.
Mardani-Kivi et al. (46)	23 patients with AAIS	Retrospective study	Arthroscopic surgery	- AOFAS scores improved from 59.21 pre-op to 88.13 at 6 months. - No significant difference in outcomes between patients with/without chondral lesions. - Effective even with chondral damage.
Ogilvie-Harris et al. (47)	17 patients with AAIS	Retrospective study	Arthroscopic surgery	- Significant improvement in pain, swelling, stiffness, and dorsiflexion. - 1 poor result (superficial infection). - 2 cases of transient foot numbness.
Ürgüden et al. (48)	41 patients with AAIS	Retrospective study	Arthroscopic surgery	- 90% excellent/good results (Meislin criteria). - Mean AOFAS: 89.6. - Poor outcomes linked to: Chondral damage (not subchondral); Post-op reinjury.

AAIS, Anterior ankle impingement syndrome; OA, osteoarthritis; AOFAS, American Orthopaedic Foot & Ankle Society Ankle-Hindfoot Scale; VAS, Visual Analog Scale; MRI, Magnetic Resonance Imaging.

Murawski et al. (31) observed a significant AOFAS score improvement from 62.83 to 91.17 (*P* < 0.001) in 41 patients with anteromedial impingement, with 93% patient satisfaction and 97% return to sports. In contrast, Jerosch et al. (32) found that only 25.7% of athletes returned to their pre-injury activity levels after partial synovectomy, suggesting worse outcomes in patients with concomitant joint laxity.

Success rates commonly exceed 80%. Tol et al. (16) reported 100% excellent outcomes in patients without osteoarthritis (OA), which declined to 77% in those with mild OA (Scranton-McDermott grade I), and noted osteophyte recurrence in two-thirds of cases. Branca et al. (33) found a 63.8% excellent rate based on McGuire score, but also reported higher recurrence in advanced OA cases (Scranton-McDermott III–IV).

Complications are uncommon (2%–7%), mainly involving transient nerve injuries (e.g., superficial peroneal nerve palsy) (30, 34). Akseki et al. (35) described two cases of postoperative neuroma-related pain, both of which were resolved with steroid injections. Arnold (36) reported excellent west point ankle scores (mean score: 86) in 81.3% of cases, although results were poorer in patients with severe cartilage damage.

### 5.2 Outcome measures

The assessment of arthroscopic outcomes for anterior ankle impingement relies on validated subjective and objective measures. For subjective evaluation, the VAS and AOFAS scores are the primary tools, showing consistent sensitivity to postoperative improvement. Yang et al. (37) reported significant AOFAS score improvement from 62.9 to 89.2 (*P* < 0.01) in patients with both anterior ankle impingement and chronic ankle instability, with results comparable to those with isolated instability. Similarly, Devgan et al. (38) observed substantial improvements in both osseous and soft tissue impingement, with AOFAS scores rising from 50.5 to 85.71 (*P* = 0.0001) and high patient satisfaction (mean Likert score 4.21/5). These findings are supported by multiple studies showing excellent functional recovery. For example, Ferkel et al. (39) reported 83.9% excellent/good outcomes at 2-year follow-up (15 excellent, 11 good out of 31 patients), with an average return to sports in six weeks. Hassan (40) observed a dramatic AOFAS improvement from 34 to 89 in 23 patients, with 95.7% of them willing to undergo the procedure again. Koczy et al. (41) further confirmed these favorable results, with AOFAS scores improving from 75.4 to 92, and only one case of transient neurapraxia among 22 patients.

Objective measures complement subjective assessments and further demonstrate surgical efficacy. Dorsiflexion range of motion (ROM) is a key functional metric. Walsh et al. (42) showed a significant improvement from 24.7° to 27.0° (*P* = 0.049) despite an 84% osteophyte recurrence rate, highlighting the disconnect between radiographic findings and clinical symptoms. Liu et al. (43) reported restored normal ankle ROM in 87% of patients. Imaging studies revealed modality-specific limitations—MRI had low sensitivity with a 40% false-negative rate for soft tissue impingement (44), whereas standard radiographs were more reliable for detecting osseous lesions (37). Large-scale analyses support these findings. Cavallo et al.'s (45) conducted a systematic review of over 600 cases, reporting 80% excellent outcomes and significant AOFAS improvement. Mardani-Kivi et al. (46) showed comparable 6-month AOFAS scores (88.13) regardless of cartilage status, reinforcing the value of arthroscopy even in complex cases with chondral pathology.

### 5.3 Long-term follow-up

Long-term studies show that the durability of arthroscopic outcomes is closely related to the degree of pre-existing joint degeneration. Tol et al. (10) demonstrated this assassination in a 6.5-year follow-up, reported sustained excellent outcomes in patients without OA, compared to only 53% excellent outcomes in those with grade II OA-notably, without progressive joint space narrowing. Walsh et al. (42) further supported long-term benefits, reporting foot functional index scores improving from 20.5 to 2.7 (*P* < 0.001) at five years, despite an 84% recurrence of osteophytes, again highlighting the disconnect between imaging and symptoms. The safety profile of arthroscopy remains favorable over time. Ogilvie-Harris et al. (47) reported durable pain relief and ROM improvement at 39 months in osseous impingement, with only one superficial infection in 17 patients. Rouvillain et al. (34) achieved 91.7% excellent outcomes using traction-free techniques in a 24-patient series, with no long-term complications. However, degenerative progression remain a concern. Baums et al. (11) reported similar 31-month outcomes (Karlsson score = 92) regardless of cartilage status. In contrast, Ürgüden et al. (48) found a significantly worse prognosis (*P* < 0.005) in patients with deep cartilage lesions at 83.7-month follow-up, especially when combined with recurrent sprains.

These findings collectively support arthroscopy as a durable and effective treatment for anterior ankle impingement, particularly in patients without advanced OA. For those with greater degeneration, adjunctive therapies may help optimize long-term outcomes (16, 33, 42).

## 6 Controversies and future perspectives in arthroscopic management of AAIS

### 6.1 Current debates in surgical management

The choice between arthroscopic and open surgical approaches for ankle arthritis remains controversial. While Lorente et al. (49) reported similar fusion rates (OR =  0.54, *P* = 0.072), Bai et al. (50) found better outcomes with arthroscopy (OR =  3.32). Arthroscopy offers clear advantages, including shorter hospital stay (MD = 2.29 days, *P* = 0.017), fewer complications (OR = 0.47, *P* = 0.016), and faster recovery times (MD = −2.31 weeks). However, open surgery may be preferred for severe cases with >5 mm osteophytes, as it achieves higher complete resection rates (92% vs. 78%). Emerging precision techniques, such as CT-guided portal placement, enhance visualization (35 ± 8°, *P* < 0.01) but require further clinical validation. Future research should focus on standardized clinical trials to optimize patient selection and assess long-term outcomes.

### 6.2 Critical limitations in current AAIS research

Although arthroscopic techniques show promise in AAIS management, four major methodological limitations reduce the reliability of current evidence. First, most studies are underpowered, retrospective or single-center series with small sample sizes (typically <100 cases), which introduces selection bias and prevents meaningful subgroup analysis by impingement type (osseous vs. soft tissue) (10, 16, 42). Second, there is substantial heterogeneity in outcome reporting. Cavallo et al. (45) identified 17 different assessment tools across 42 studies, ranging from validated scales like AOFAS to subjective surgeon-based ratings (e.g., “excellent/good” ratings). Third, long-term efficacy data are limited-only 18.6% of studies include ≥5-year follow-up (10, 42, 48), leaving key questions about postoperative osteoarthritis progression unanswered. Fourth, temporal bias is a major concern in the current literature. Only 12.5% (3/24) of the included studies were published after 2016, despite significant advancements in arthroscopic and open surgical techniques during that time. This gap may lead to under-representation of current success rates or complication profiles, especially for advanced techniques like robotic-assisted debridement. As a result, caution is warranted when applying these findings to current practice, emphasizing the need for updated systematic reviews incorporating post-2016 evidence. To address these limitations, coordinated multi-center RCTs are needed, incorporating: (1) standardized outcome measures, (2) at least 5 years of follow-up using structural MRI and functional endpoints, and (3) economic evaluations to assess value-based adoption thresholds.

## 7 Summary

Arthroscopic surgery is the current standard of care for AAIS, supported by level I evidence showing better patient-reported outcomes, faster functional recovery, and fewer complications compared to open techniques. Its main advantages include precise removal of osseous and soft tissue lesions, minimal disruption to joint biomechanics, and lower perioperative morbidity. However, achieving optimal outcomes requires a multidisciplinary approach, including dynamic preoperative imaging, personalized surgical planning, and structured rehabilitation.

Despite these advances, important gaps remain, particularly regarding long-term degenerative changes and the cost-effectiveness of new technologies. Furthermore, comparative effectiveness studies should address socioeconomic disparities in access to these emerging treatment options. The orthopedic community must prioritize rigorous long-term studies to improve surgical decision-making and ensure value-based care for AAIS patients across diverse populations.
